# Hydrothermal reaction time-dependent morphology and electrochemical performance of BaMoO_4_ for asymmetric supercapacitors

**DOI:** 10.1371/journal.pone.0354958

**Published:** 2026-09-29

**Authors:** S. Sakthivel, Manikandan Ayyar, B. Kabilan, D. Shanmugapriya, Z. Mohamed Riyas, B. Preethi, B. Archana, Kartikey Verma, Asefash Getachew Girma

**Affiliations:** 1 Department of Chemistry, Karpagam Academy of Higher Education, Coimbatore, Tamil Nadu, India; 2 Centre for Material Chemistry, Karpagam Academy of Higher Education, Coimbatore, Tamil Nadu, India; 3 Vicerrectoría de Investigación y Postgrado, Universidad de La Serena, La Serena, Chile; 4 Department of Physics, Centre for Material Chemistry, Karpagam Academy of Higher Education, Coimbatore, Tamil Nadu, India; 5 Department of Chemistry, Faculty of Engineering, Karpagam Academy of Higher Education, Coimbatore, Tamil Nadu, India; 6 Centre for Interdisciplinary Research and Innovation, Krishna Institute of Engineering & Technology (KIET), Ghaziabad, Uttar Pradesh, India; 7 Department of Chemical Engineering, Mattu University, Mettu, Ethiopia; National Chung Cheng University College of Engineering, TAIWAN

## Abstract

The escalating global energy crisis necessitates the development of sustainable and high-performance energy storage systems. In the present work, the barium molybdate (BaMoO_4_, BMO) nanoparticles were prepared using a hydrothermal process for different reaction times (5, 10, 15, and 20 h) and the formation process and its effect on the electrochemical behaviour were systematically studied. The structural and vibrational analyses showed that the pure tetragonal phase BMO-15 with an average crystallite size value of 17.05 nm. The characteristic Ba–O and Mo–O bonding was confirmed by Fourier-transform infrared (FTIR) spectroscopy, and the high-resolution transmission electron microscopy (HRTEM) confirmed the presence of uniformly distributed spherical nanoparticles. The time-dependent samples were analysed using field-emission scanning electron microscopy (FESEM), which revealed a systematic change in the morphology, from the initial nuclei, to the well-developed crystalline nanoparticles with the reaction time, thereby giving an insight into the growth mechanism. The optimized BMO-15 electrode showed superior specific capacitance of 531 F g^−1^ at the current density of 1 A g^−1^ with retaining 90% capacity after 10,000 charge–discharge cycles at 6 A g^−1^, which revealed its cycling stability. Moreover, the fabricated asymmetric supercapacitor device (BMO-15//AC) exhibited a remarkable energy density of 33.05 Wh kg^−1^ and power density of 1399 W kg^−1^, and it maintained 72% of its initial capacitance after 5000 charge-discharge cycles. The electrochemical performance is explained by the structural stability of the tetragonal BMO-15 framework, the high redox activity of the active sites of Mo and the synergistic effect of the BMO-15 and activated carbon electrodes. Based on these results, BMO-15 nanoparticles are shown to be a promising material for the next generation of high-performance supercapacitors which can be considered as a sustainable material.

## 1. Introduction

Supercapacitors have become a source of great interest because of their capabilities to provide efficient and reliable energy storage systems, especially with the rapid evolution of energy storage technologies, to satisfy the increasing global demand for such. The transition to sustainable and clean energy has driven research to develop materials and devices that are capable of meeting high energy density and high-power density requirements [1,2]. Conventional lithium-ion batteries are distinct in terms of their properties, including higher energy density, slower charge/discharge cycles, and less power delivery. The characteristics make them suitable for the next generation of energy storage and hybrid energy systems [3]. These benefits are reduced manufacturing costs, high energy density, high-rate charging/discharging, long life, excellent coulombic efficiency, and low internal resistance. These enhance the operations of modern energy systems, including the ability to power electric vehicles, portable devices and hybrid energy systems.

Supercapacitors have several unique benefits over conventional battery storage technologies in terms of performance, longevity, and energy delivery efficiency. Batteries, on the other hand, are prized for their high energy density, long service life and ability to power renewable energy sources, and for their ability to charge and discharge rapidly. However, due to their lower energy density, supercapacitors are limited. This restriction emphasises the requirement to research ways of enhancing their energy density both in terms of cell voltage (V) and specific capacitance (Cs). The specific capacitance of the electrode can be increased by increasing the surface area, modifying the porosity and enhancing the electrical conductivity of the electrode. Redox-active materials can be added to the electrolyte and the electrodes designed to provide an extended potential window [3]. There are two types of supercapacitors, based on their energy storage mechanisms, namely, electrical double-layer capacitors (EDLCs) and pseudocapacitors (PSCs). The mechanism of energy storage in EDLCs is based upon three hypotheses: Helmholtz, Gouy–Chapman, and Stern theories. These theories describe the development of an electric field at the electrolyte-electrode interface, which allows energy to be stored by electrostatic adsorption. Among the carbon-based materials, graphene oxide (GO), activated carbon (AC), carbon nanotubes (CNTs) and carbon quantum dots (CQDs) are the most promising candidates, in this context, because of their high surface area and remarkable electrical conductivity, which facilitate effective ion adsorption and superior charge storage capacities [4,5].

In contrast, pseudocapacitors store energy by reversible fast faradaic redox reactions with the electrode–electrolyte interface during charging and discharging. Various transition metal oxides, hydroxides, sulfides and conducting polymers have been studied as electrode materials due to their ability to change the valence state in their materials to increase the redox activity. These faradaic reactions are very important for increasing specific capacitance and energy density of pseudocapacitors as compared to EDLCs. However, both EDLCs and pseudocapacitors experience a number of key limitations, including relatively low energy density, ion diffusion limitation, and long-term instability. To overcome such problems, hybrid supercapacitors (HSCs), which are composed of EDLC and pseudocapacitive materials, have been developed, integrating non-faradaic and faradaic charge storage mechanisms. By using this combination approach, HSCs could have better capacitance, higher energy density, and higher operational voltage compared with the systems of a single capacitor [6–9]. Supercapacitors (SCs) have high maximum power density, but lower energy density, which is a challenge for broader applications. There are several strategies that can be effectively applied to overcome this limitation. For example, one common method is to improve the specific capacitance of electrode materials via structural modifications, as well as to increase the voltage window by creating asymmetric supercapacitor devices. The objective of these strategies is to achieve high power density along with higher energy density. There are three types of configurations: symmetric (two electrodes with the same mechanism), asymmetric (two electrodes with different mechanisms) and hybrid (two different electrodes with different mechanisms). At present, hybrid-type supercapacitors are preferred because of their higher efficiency. Their specific capacitance is higher due to their asymmetric configuration, and they are favoured because they can increase energy density without sacrificing power density. The asymmetric configuration offers several electrochemical benefits to hybrid supercapacitors, such as increased charge separation distance, enhanced redox kinetics, expanded surface-active area, and effective coupling of faradaic and non-faradaic processes. For this reason, the use of asymmetric supercapacitors (ASCs) as a viable compromise between EDLCs and pseudo-capacitors, where a pair of dissimilar electrodes store energy by different mechanisms, has been proposed. In the context of the various electrode materials, transition metal oxides, sulfides, hydroxides and nitrides have been studied extensively, as they are known to have tunable valence states and high theoretical capacitance, and they exhibit remarkable electrochemical reversibility [10–12]. Among the various transition metal oxides, Co_3_O_4_, MnO_2_, RuO_2_, NiCo_2_O_4_, and ZnCo_2_O_4_ have exhibited excellent pseudocapacitive properties and they are all characterised by high electrical conductivity, rich redox activity and versatile structure. In this class, scheelite-type molybdates (AMoO_4_; A = Ba, Sr, Co, Ni, Pb, etc.) have recently gained much interest as high-performance supercapacitor electrode materials. These features of their stable crystal structure, multiple redox centres, and effective ionic diffusion pathways along the crystal lead to increased charge storage capacity and long-life electrochemical stability [13,14].

The barium molybdate (BMO, BaMoO_4_) among these is remarkable for its well-defined tetragonal crystal structure and more than one oxidation level of molybdenum and for its rapid ion transport properties. These characteristics make the material able to undergo faradaic reactions in a very fast and reversible manner, thereby securing high specific capacitance and cycling life. Furthermore, BaMoO_4_ has other merits such as environmental benignity, cost-effectiveness, and high chemical durability, making it a potential material for the next generation of energy storage devices [15–17]. It has been found recently that its electrochemical properties can be further improved by doping and making composites of BaMoO_4_ with other elements or phases. For instance, Sm doped BaMoO_4_ was found to have a capacitance of 135 F g^−1^ after 5000 cycles with 83.3% retention [18], Sr-doped BaO nanorods showed a capacitance value as high as 925.2 F g^−1^ [19], and Ag/BaMoO_4_ nanoparticles showed a capacitance value as high as 443 F g^−1^ [20]. The results showed that the material based on BaMoO_4_ has a high potential for electrochemical energy storage. Nevertheless, although the above-mentioned progresses have been achieved in the studies of pristine BaMoO_4_ nanoparticles and their asymmetric device configuration, a further exploration is still needed to explore their full electrochemical potential [21].

The material was synthesised by the hydrothermal method and characterised in detail as a potential high-performance supercapacitor electrode material, as the objective of this study is to develop such a material with BMO. The formation of BMO was further confirmed by standard physicochemical characterisation. An enhanced specific capacitance of 531 F g^−1^ at 1 A g^−1^ was realised for the BMO-15 electrode, and it retained 90% of its initial capacitance following 10,000 charge–discharge cycles at 6 A g^−1^, showing very good cycling stability and durability. Further, the asymmetric supercapacitor device fabricated here (BMO-15//AC) showed excellent electrochemical performance with higher energy and power densities compared to many other reported molybdate-based asymmetric supercapacitors. The findings highlight the promise of BMO-15 as an electrode material for future energy storage applications, and suggest a straightforward method that can be followed to improve other transition-metal molybdates for optimal supercapacitor performance.

## 2. Experimental

### 2.1. Chemicals/Reagents

Urea, potassium hydroxide (KOH), ammonium molybdate tetrahydrate, and barium nitrate hexahydrate [Ba(NO_3_)_2_·6H_2_O] of ≥99.9% purity were purchased from Sigma-Aldrich, while nickel foam was obtained from Vritra Technologies. All chemicals were of analytical grade and used without any further purification.

### 2.2. Preparation of barium molybdate

BaMoO_4_ (BMO) were produced using a straightforward one-pot hydrothermal technique. In a standard procedure, 0.1 M barium nitrate [Ba (NO_3_)_2_·6H_2_O], 0.1 M ammonium molybdate tetrahydrate [(NH_4_)_6_Mo_7_O_24_·4H_2_O], and 0.4 M urea were dissolved in 70 mL of deionised (DI) water while being stirred continuously until a clear solution. The prepared precursor solution, which was then transferred into a Teflon-lined stainless-steel autoclave and sealed. The hydrothermal reaction proceeded at 160 °C at 15 h in a vacuum oven. Other samples were prepared with the hydrothermal times of 5, 10, and 20 h, respectively. The resulting product was thoroughly rinsed multiple times with DI water and ethanol, and subsequently dried in a hot-air oven at 60 °C overnight. In the final step, the dried sample was calcined at 400 °C for 2 hours. The final products were marked as BMO-5, BMO-10, BMO-15, and BMO-20, which were then utilised for structural and electrochemical analysis. Fig 1 illustrates the Hydrothermal Synthesis of BMO.

**Fig 1:**
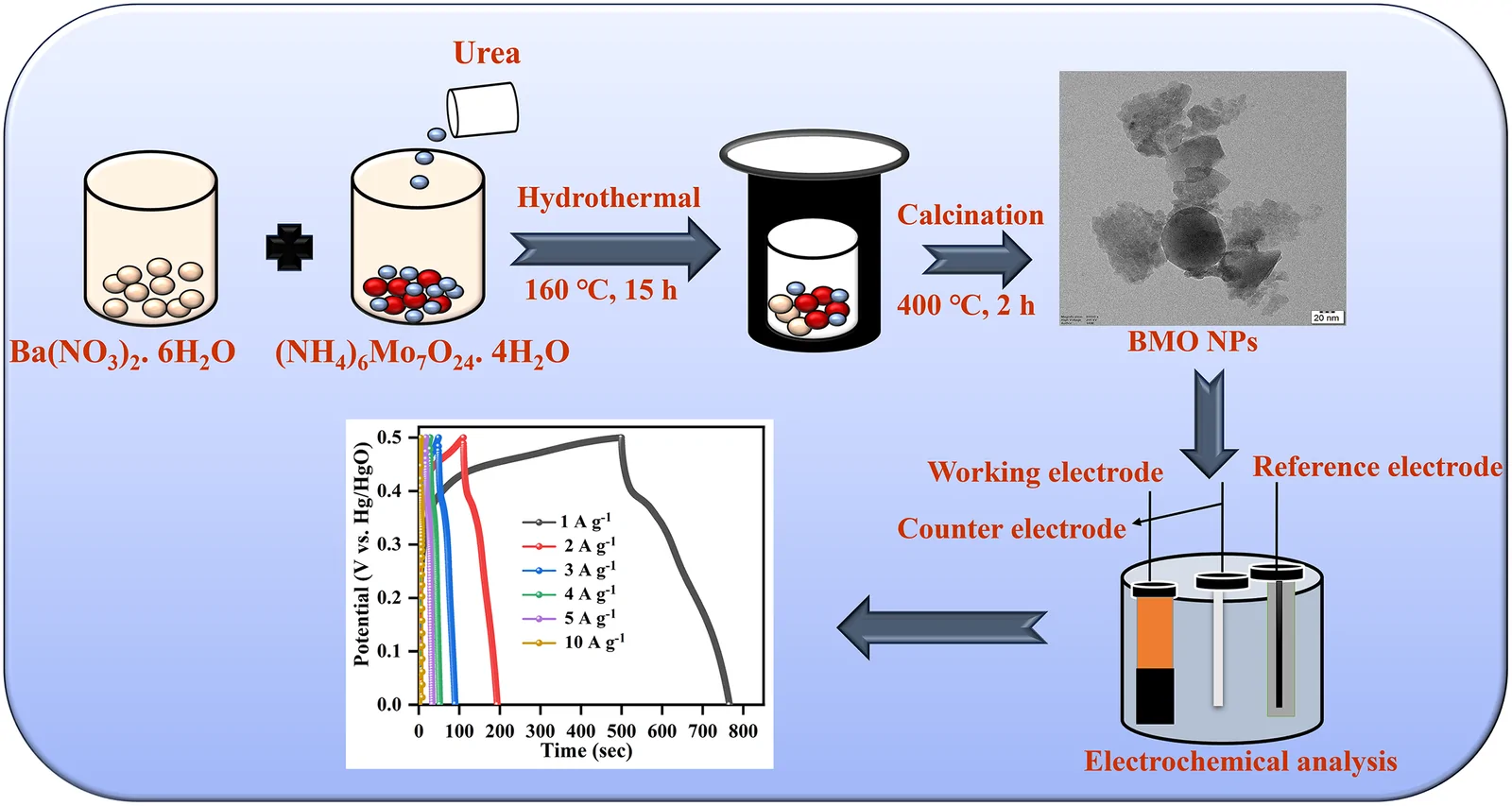
Schematic Illustration of the Hydrothermal Synthesis of BMO.

### 2.3. Physical measurements

The phase purity and structure of the obtained sample (Rigaku Ultima Mini Flex) were examined using the XRD technique, which uses Cu Kα radiation at (λ = 1.5418 Å). An FT-IR spectrum (Perkin-Elmer) was recorded to confirm the functional group of the samples. The morphology of the samples was characterized by FESEM (Thermo Scientific Apreo S and Carl Zeiss Gemini 300). The BET surface area and BJH pore size distribution were measured using a BELSORP MAX analyzer. A HRTEM (JEM-2100 Plus) was used for the morphology analysis of the samples. The electrochemical measurements for EIS, GCD, and CV of the produced electrode were evaluated using a CHI660E system.

### 2.4. Fabrication of the electrode

Prior to electrode preparation, nickel foam was ultrasonically cleaned with ethanol and deionised water, then dried thoroughly. To prepare the working electrodes the active material was coated onto cleaned nickel foam with a homogeneous slurry of the active material, the activated carbon and polyvinylidene fluoride (PVDF) in a 80:10:10 weight ratio with a solvent of N-methyl-2-pyrrolidone (NMP). The coated electrodes were then dried overnight in an oven at 80 °C to remove all the solvent content. The electrochemical properties were studied in a three-electrode cell with an electrolyte of 6 M KOH solution. In this configuration, the prepared BMO electrode was used as the working electrode, a mercury/mercury oxide (Hg/HgO) electrode as the reference electrode and a platinum (Pt) wire as the counter electrode. The electrochemical behaviour of the electrode materials was characterised by cyclic voltammetry (CV), galvanostatic charge– discharge (GCD) and electrochemical impedance spectroscopy (EIS) measurements. Specific capacitance (C_s_) of working electrode was determined from either of CV profiles or GCD profiles as follows:


$$\begin{array}{c} C_{s_{P_{\text{1}}}}=\frac{\int I(\nu )dV}{m\times V}\;(\text{F}/\text{g}) \end{array}$$ (1)

$$\begin{array}{c} C_{s_{P_{\text{2}}}}=\frac{I\times \Delta t}{m\times \Delta v}\;(\text{F}/\text{g}) \end{array}$$ (2)

where C_sp1_ and C_sp2_ are specific capacitances derived from the CV and GCD analyses, respectively. I(V) is the current response at the sweep voltage V, v is the scan rate (mV s^−1^), ΔV is the potential window (V), I is the applied current (A), Δt is the discharge time (s), and m is the mass (g) of the active material loaded onto the Ni-foam current collector.

### 2.5. Asymmetric BMO//AC device fabrication

The asymmetric supercapacitor device was fabricated with activated carbon serves as the negative electrode, BMO-15 serves as the positive electrode, and the Whatman filter paper was used to separate the two electrodes, and the 6 M KOH/PVA gel was employed as the electrolyte. The asymmetric supercapacitor device energy and power density were determined using the following formulas.


$$\begin{array}{c} E=\frac{C_{dev}{\times (\Delta V)}^{\text{2}}}{\text{2}\;\times \text{3}.\text{6}}\;(\text{Wh}/\text{kg}) \end{array}$$ (3)

$$\begin{array}{c} P=\;\frac{\text{3}\text{6}\text{0}\text{0}\;\times E}{\Delta t}\;(\text{W}/\text{kg}) \end{array}$$ (4)

The coulombic efficiency was calculated to the following equation:


$$\begin{array}{c} \eta =\frac{t_{d}}{t_{c}}\times \text{1}\text{0}\text{0} \end{array}$$ (5)

where t_c_ and t_d_ are the charging and discharging time of the capacitor using cyclic stability.

## 3. Results and discussions

### 3.1. XRD analysis

X-ray diffraction (XRD) was employed to confirm the phase purity of BMO-15. The XRD pattern of the BMO-15 is shown in Fig 2. The 2θ values of 26.27°, 28.16°, 32.20°, 36.95°, 41.93°, 42.89°, 45.96°, 48.57°, 53.81°, 67.34°, 69.23°, and 76.58° correspond to the diffraction planes (112), (004), (200), (211), (213), (204), (220), (116), (224), (208), (316), and (228), respectively. These diffraction peaks are consistent with JCPDS card number 00-029-0193 [22], indicating that the BaMoO_4_ nanoparticles were synthesised without the presence of any detectable impurity phases. The trend is similar to that of the tetragonal crystal structure of BMO-15. The crystallite size of the BMO nanoparticles was calculated by the Debye–Scherrer formula:

**Fig 2:**
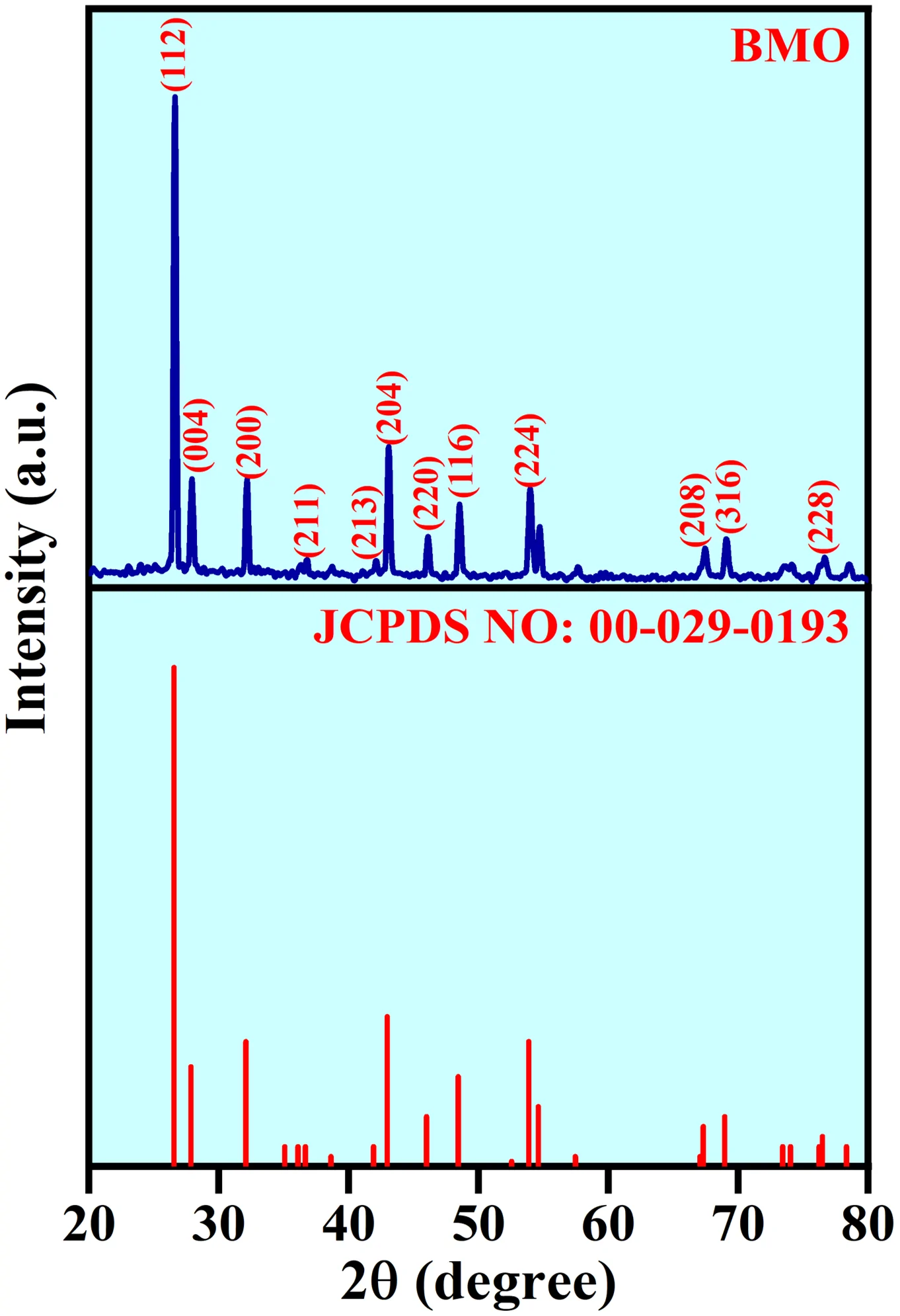
XRD pattern of BMO- 15.


$$\begin{array}{c} D\;=\;K\lambda \;/\;(\beta \;cos\theta ) \end{array}$$ (6)

The shape factor K is 0.94, the X-ray wavelength is λ = 1.5418 Å (for Cu Kα), the full width at half maximum (FWHM) of the diffraction peak is β (in radians), and the Bragg angle is θ. The crystallite size of the sample was calculated to be 17.05 nm [23]. The crystallite size decreases the surface area and access of ions to the electrode creating faster charge/discharge cycles and higher cycling stability, thereby improving the performance of the supercapacitor.

### 3.2. FT-IR analysis

The FT-IR spectrum of the BMO-15 (Fig 3) shows characteristic absorptions at 1688, 963, 786 and 455 cm^−1^. The weak band at 1688 cm^−1^ is assigned to the H–O–H bending mode of surface adsorbed water which represents a trace amount of moisture. The formation of BaMoO_4_ is confirmed by the very sharp and intense band at 963 cm^−1^, which is due to the symmetric stretching vibration of the Mo–O bond in the MoO_4_^2−^ tetrahedral units. The bands at 786 cm^−1^ and 455 cm^−1^ are due to the asymmetric stretching and bending of Mo–O–Mo linkages, respectively, further confirming the scheelite-type crystal structure. The well-resolved vibrational features confirm the strong and stable nature of the crystal structure in BaMoO_4_, which promotes efficient electron transport and swift ions diffusion [24].

**Fig 3:**
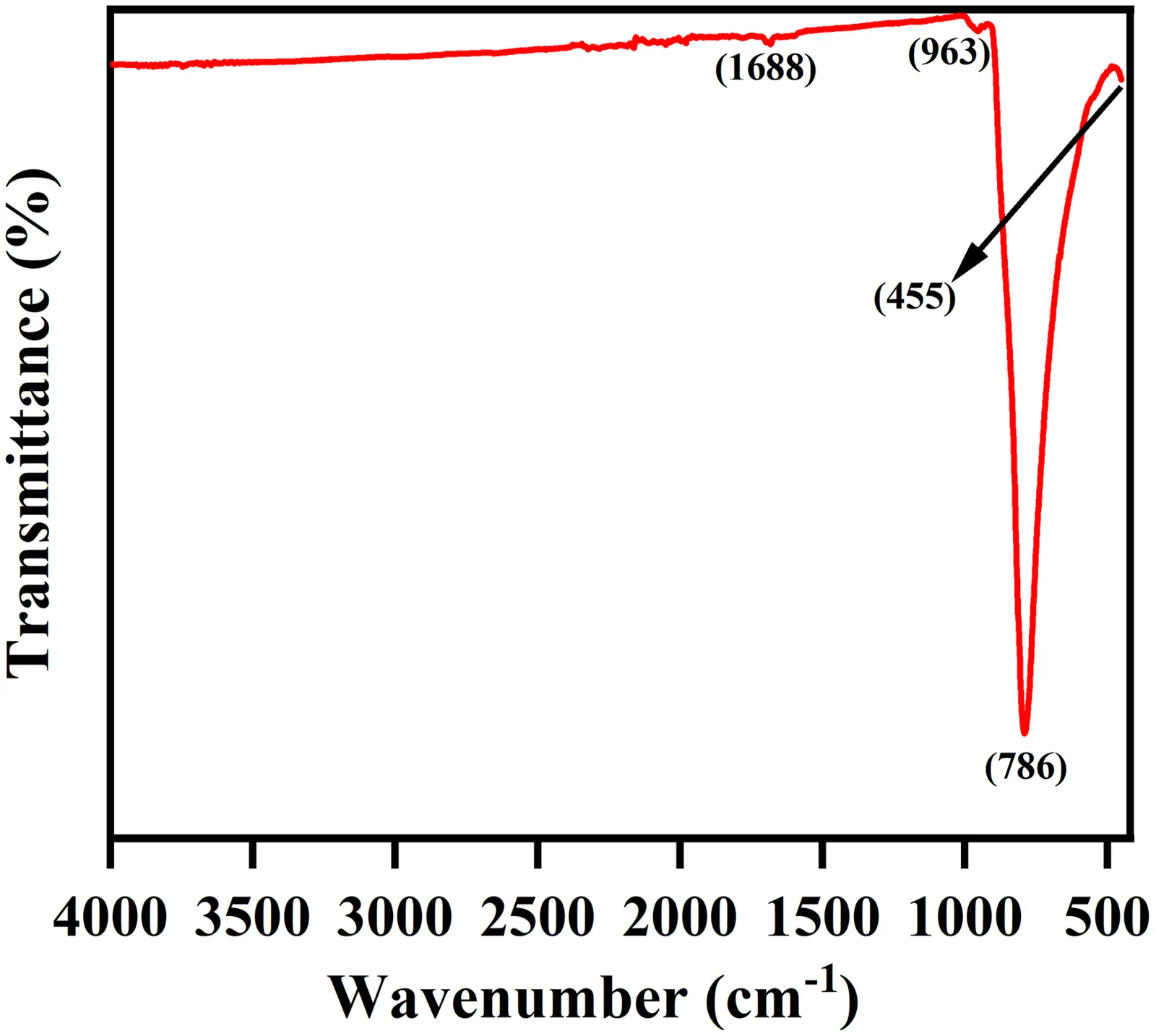
FT-IR spectra of BMO-15.

### 3.3. FESEM analysis

Surface morphology of the samples BMO-5, BMO-10, BMO-15 and BMO-20 were described using FESEM. All samples showed the same structure of a nanoparticle, observed from the images of BMO-5, BMO-10, and BMO-20, as presented in S2, S3 and S4 Figs in S2 File respectively. The BMO-15 sample showed the nanosheet-like structure depicted in Fig 4(a, b) as the hydrothermal duration increased, EDS (Fig 4c) and elemental mapping was conducted. In Fig 4(d-g), the elemental mappings are displayed for barium (Ba), molybdenum (Mo) and oxygen (O). The SEM images show the interfacial integration resulting in the formation of a hybrid structure that exhibits enhanced electrochemical surface area and electronic conductivity.

**Fig 4:**
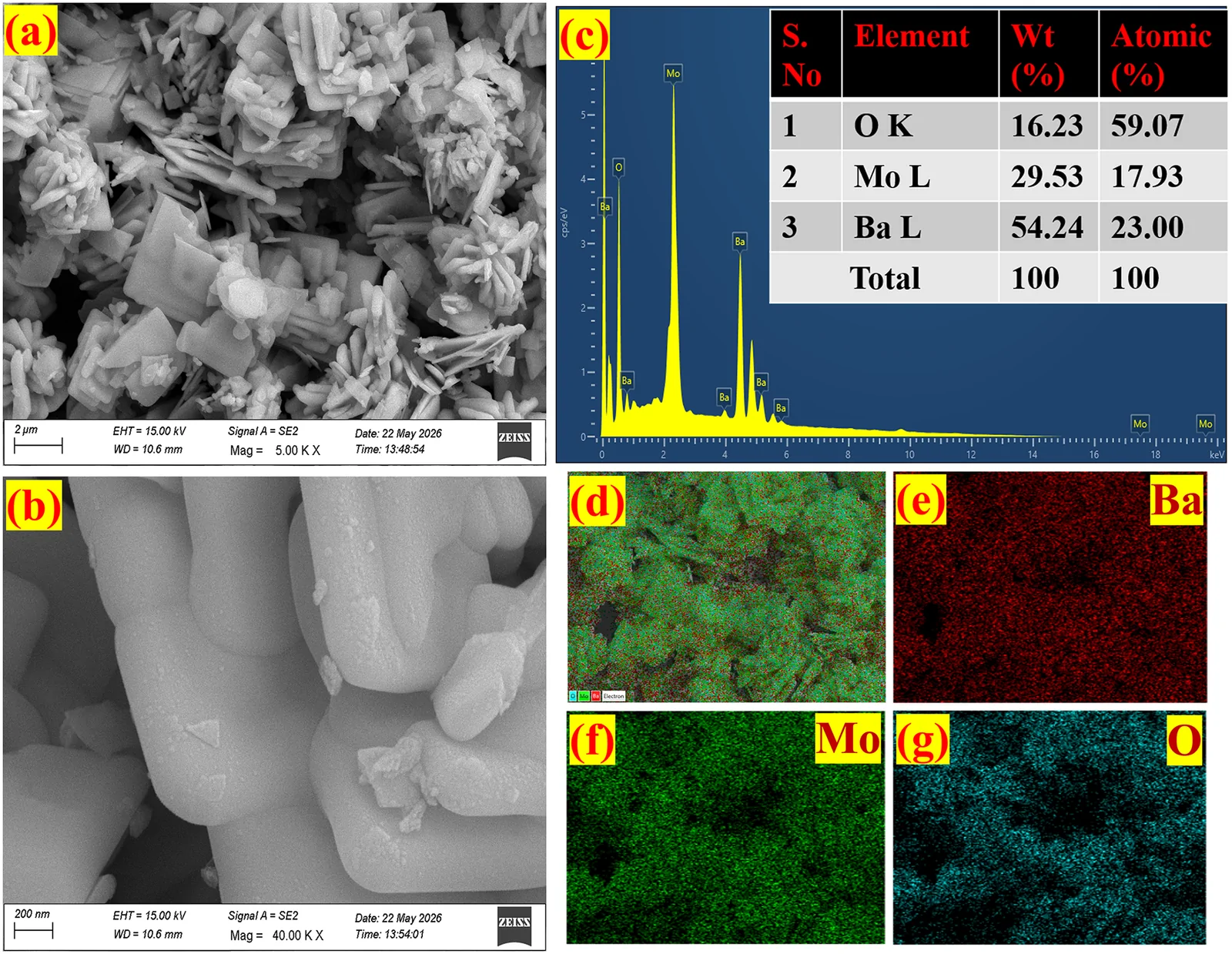
(a, b) FESEM images of BMO-15, (c) EDS spectrum of BMO-15, (d-g) Element mapping images of Ba, Mo, O.

### 3.4. HRTEM & SAED analysis

As indicated in Fig 5(a) and 5(b) the BMO-15 nanoparticles have a spherical shape with some agglomeration and their size is in the range of 20–50 nm. The high resolution HRTEM image (Fig 5c) shows clear lattice fringes, which have an interplanar spacing of 0.423 nm, matching the (112) plane of the tetragonal BaMoO_4_ phase. In addition, selected area electron diffraction image (Fig 5d) shows clear concentric rings, indicative of a polycrystalline nature of the material. This result is in agreement with the XRD result, which showed that the nanostructured BaMoO_4_ was formed in a phase-pure manner.

**Fig 5:**
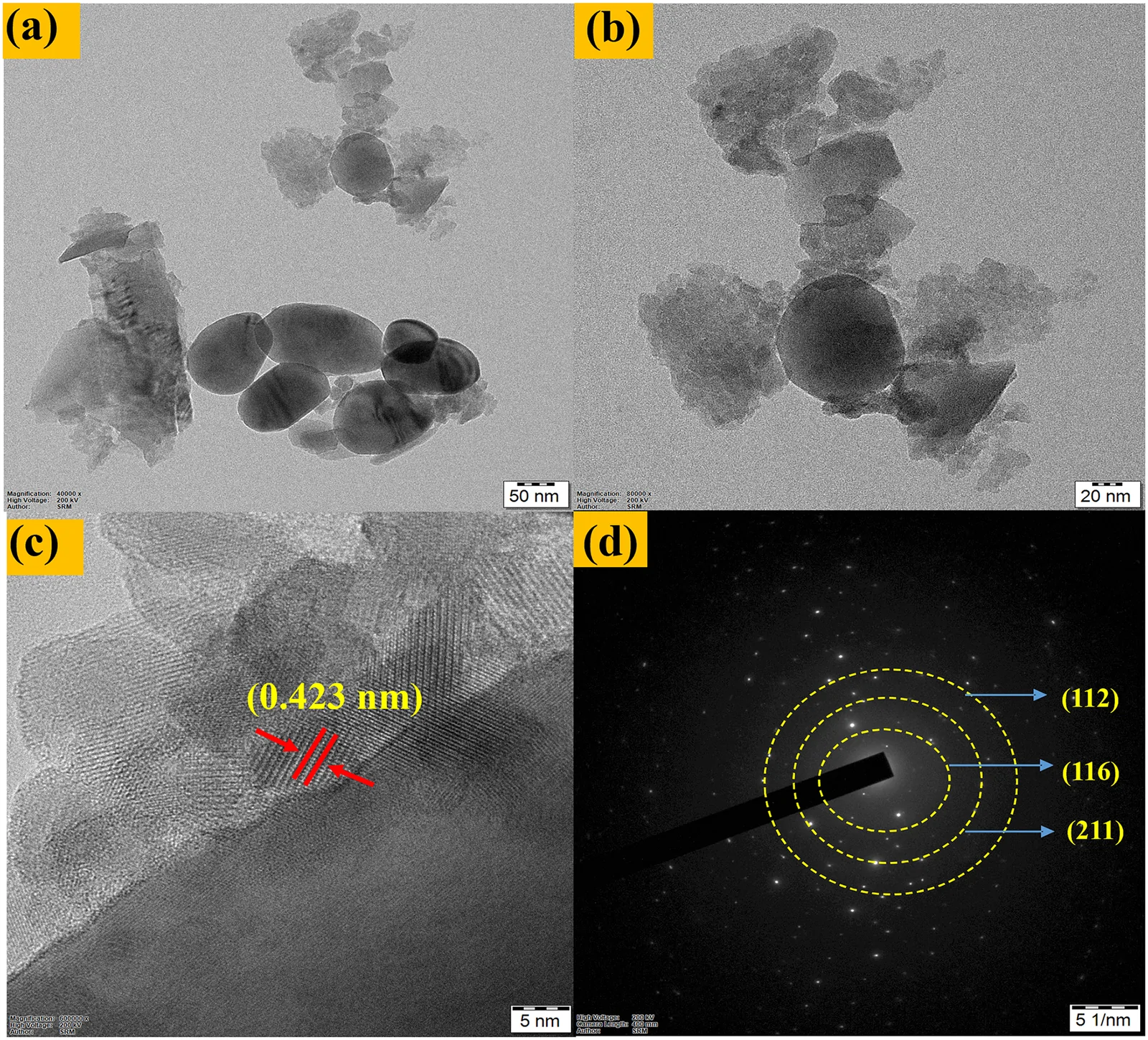
TEM images (a, b), HR-TEM image (c), and SAED pattern (d) of BMO-15.

### 3.5. BET analysis

The N_2_ adsorption/desorption isotherms of the synthesised BMO-15 electrode material is displayed in Fig 6(a). It is observed that at low P/P₀ the isotherm shows a gradual increase of adsorption of N_2_, and at high relative pressure (P/P₀ = 1.0) it shows sharp uptake, which suggests the presence of mesoporous characteristics. The mesoporous structure is very advantageous for supercapacitors because it enables the electrolytic ion diffusion to be fast and acts to enhance the charge transfer kinetics in the electrochemical process. The BJH pore size distribution curve for the BMO-15 sample is shown in Fig 6(b), which indicates the presence of a wide range of pore diameters, with a large number of nanoscale mesopores. The BET analysis showed a specific surface area of 2.56 m² g^-1^ and a pore volume of 0.013 cm³ g^-1^. The porous architecture and interconnected pore channels ensure the presence of plenty of electroactive sites and increase the accessibility of the electrolyte onto the surface of the electrode. Thus, the mesoporous structure of BMO-15 plays a significant role in showing improved ion transport, good capacitive behaviour, and electrochemical performance for the application of supercapacitors.

**Fig 6:**
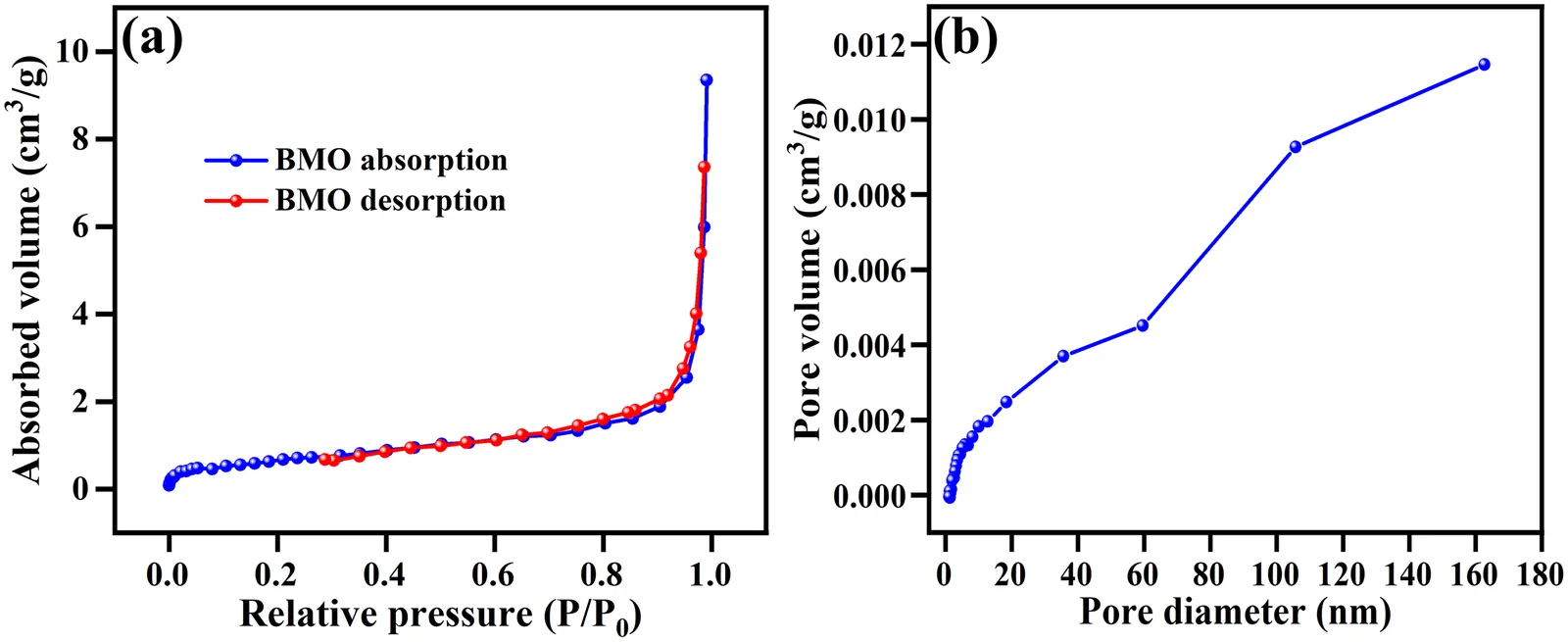
(a) N_2_ adsorption-desorption isotherms and (b) Pore size distributions of BMO-15.

### 3.6. Electrochemical analysis

The cyclic voltammetry (CV) curve of BMO-15 was shown in Fig 7(a) which was recorded at the potentials between 0–0.5 V with a scan rate ranging from 2 to 100 mV s^−1^. The CV profile of the BMO-15 displays significantly larger enclosed area than BMO-5, BMO-10, and BMO-20, suggesting that it has much better charge storage properties, and also has well-defined redox peaks. The better the area under the CV profile means the better the charge separation. CV profiles of the BMO-5, BMO-10 and BMO-20 electrodes are given in the S4a, S5a, and S6a Figs in S2 File. The differences are due to the different charge storage mechanisms and different charge transfer kinetics of the two electrodes [25]. The combination of barium and molybdate species results in a synergistic interaction, which is helpful for the improvement in electrochemical properties of BMO-15. The nonlinear CV curves validate the pseudocapacitive behaviour of the electrode, with a high level of reversibility and large capacitance. Specific capacitance (C1) values of 2000, 1206, 824, 293, 186, and 93 F g^−1^ were obtained for BMO-15 at scan rates of 2, 5, 10, 20, 50, and 100 mV s^−1^, respectively. Fig 7(b) shows that the anodic and cathodic peaks move to more anodic and cathodic potentials, respectively at higher scan rates because of the limited diffusion of ions, which slows the electrochemical neutralisation process during the redox process. The charge storage mechanism can be written as:

**Fig 7:**
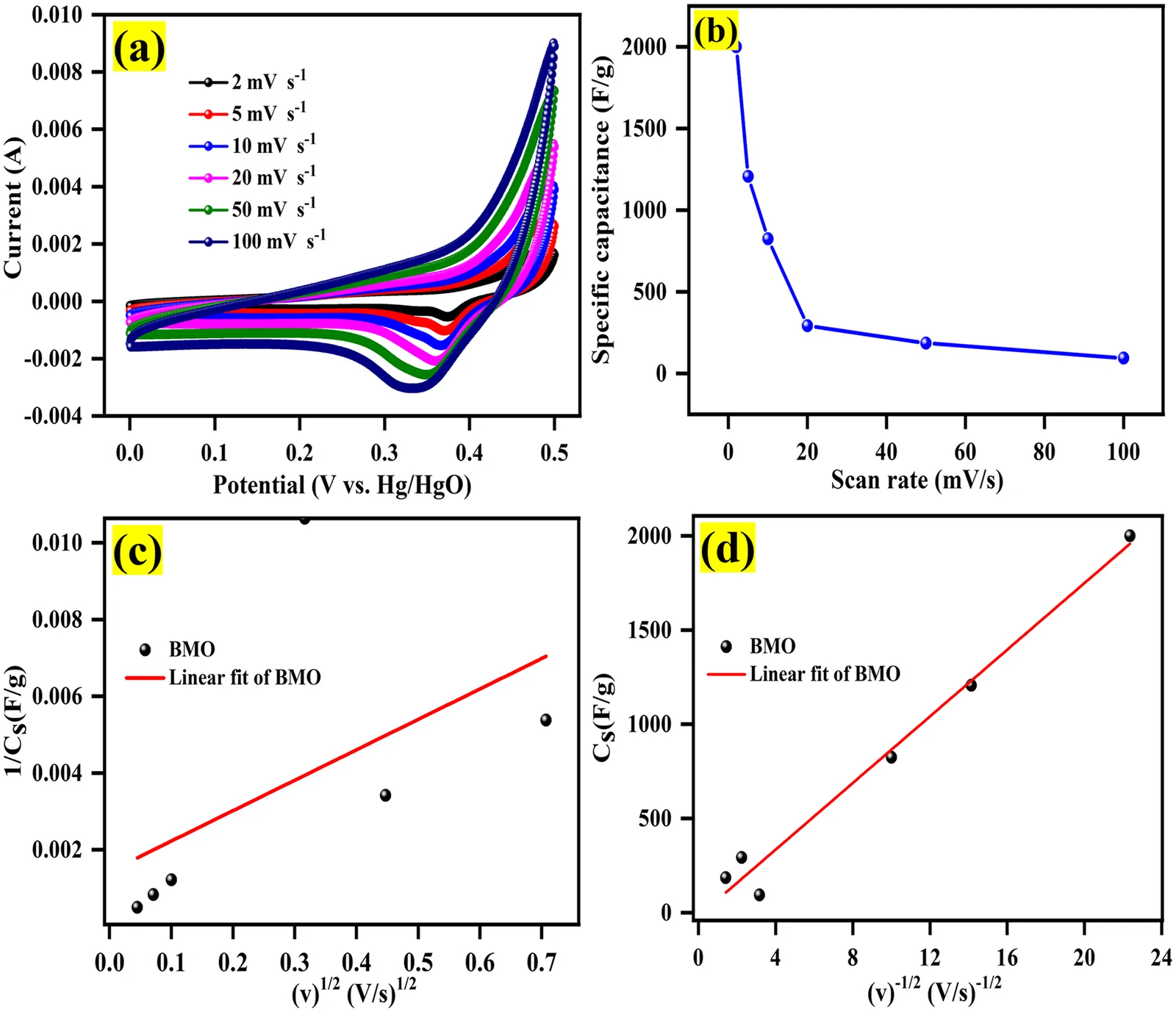
(a) CV spectra, (b) Scan rate vs capacitance, (c) Total Csp (v^1/2^ vs Cs^-1^), and (d) outer Csp (v^-1/2^ vs Cs) of BMO-15.


$$\begin{array}{c} \text{BM}\;+\;{\text{OH}}^{-}\;⇌\;\text{BM}(\text{OH})\;+\;{\text{e}}^{-} \end{array}$$ (7)

$$\begin{array}{c} \text{BMO}\;+\;{\text{OH}}^{-}\;⇌\;\text{BMOOH}\;+\;{\text{e}}^{-} \end{array}$$ (8)

The pseudo-capacitive contribution is mainly due to the faradaic redox reactions at the electrode–electrolyte interface. The successive and reversible faradic oxidation and reduction of Mo involve an array of oxidation states:


$$\begin{array}{c} {\text{Mo}}^{\text{6}+}⇌\;{\text{Mo}}^{\text{5}+}⇌\;{\text{Mo}}^{\text{4}+}⇌\;{\text{Mo}}^{\text{3}+} \end{array}$$ (9)

The overall capacitance can be accounted for by these reversible electron transfer events. The capacitive and diffusion contributions were separated from each other in the Trasatti method, which was applied with the following equations [29]:


$$\begin{array}{c} q(\nu )\;=\;k/\sqrt{\nu }\;+\;q_{0} \end{array}$$ (10)

$$\begin{array}{c} \text{1}/q(\nu )\;=\;k\sqrt{\nu }\;+\;\text{1}/q_{T} \end{array}$$ (11)

Plots of √v versus 1/q and of 1/√v versus q were made by extrapolating to the limits ν → 0 and ν → ∞, respectively, as shown in Fig 7(c), to find the total capacitance (q_T_) and outer capacitance (q_0_). The total capacitance (q_T_) and outer capacitance (q_0_) were found to be 2000 F/g and 93 F/g, respectively, as shown in Fig 7(d) [26,27]. The outer capacitance (C_outer_) is the charge stored at the outermost surface of the working electrode and the total charge (C_total_) is the sum of the outer layer (C_outer_) and the inner layer (C_inner_).


$$\begin{array}{c} C_{\text{total}}\;=\;C_{inner}\;+\;C_{outer} \end{array}$$ (12)

The amount of charge stored capacitively and the amount stored in the diffusion controlled process were determined using:


$$\begin{array}{c} q_{\text{i}}\;(\%)\;=\;(q_{\text{i}}\;/\;q_{T})\;\times \;\text{100}\% \end{array}$$ (13)

$$\begin{array}{c} q_{\text{o}}\;(\%)\;=\;(q_{\text{o}}\;/\;q_{T})\;\times \;\text{100}\% \end{array}$$ (14)

The galvanostatic charge–discharge (GCD) curves of BMO-15 electrode obtained at current densities of 1, 2, 3, 4, 5 and 10 A g^−1^ over the potential range of 0–0.5 V are shown in Fig 8(a). This is seen in Figs. that the BMO-15 electrode had a longer discharge time as compared to BMO-5, BMO-10 and BMO-20 at 1 A g^−1^. Shown in S4b, S5b and S6b Figs in S2 File respectively. The specific capacitance of BMO-15 reaches 531 F g^−1^, surpassing those of BMO-5 (45 F g^−1^), BMO-10 (38 F g^−1^), and BMO-20 (49 F g^−1^) (S4c, S5c, and S6c Figs in S2 File). The discharge curves are asymmetric and non-linear, a characteristic that is indicative of the significant faradaic redox process of the synthesised material. The window was designed with great care, so as not to create too much polarization and undesirable voltage drop. As shown in Fig 8(b), the BMO-15 electrode delivers specific capacitance values of 531, 333, 245, 191, 156, and 81 F g^−1^ at current densities of 1, 2, 3, 4, 5, and 10 A g^−1^, respectively. A comparative analysis of various synthesis methods reported for barium-based nanoparticles is summarised in Table 1. The internal resistance (IR drop) is determined by the electrical conductivity of the KOH electrolyte, its ionic mobility and resistance of electrodes. As the current density increases, the time for the ions to travel through the active material becomes shorter and the redox reactions at the electrode/electrolyte interface further restrict the ion transport. This results in a decrease in the capacitance as the current density increases, since there is a lack of faradaic activity at high current densities [28].

**Table 1:** Comparison analysis of various synthesis methods for barium-based nanoparticles.

S. No	Materials	Method	Current density	Specific capacitance	Ref.
1	Ag/BaMoO_4_	Microwave method	1 A g ^− 1^	443 F g ^− 1^	[20]
2.	Sm-doped BaMoO_4_	Co-precipitation method	1 A g ^− 1^	135 F g ^− 1^	[18]
3	Barium oxide	SILAR method	–	405.72 F g ^− 1^	[31]
4	BaMoO_4_	Hydrothermal method	1 A g ^− 1^	531 F g ^− 1^	**This work**

**Fig 8:**
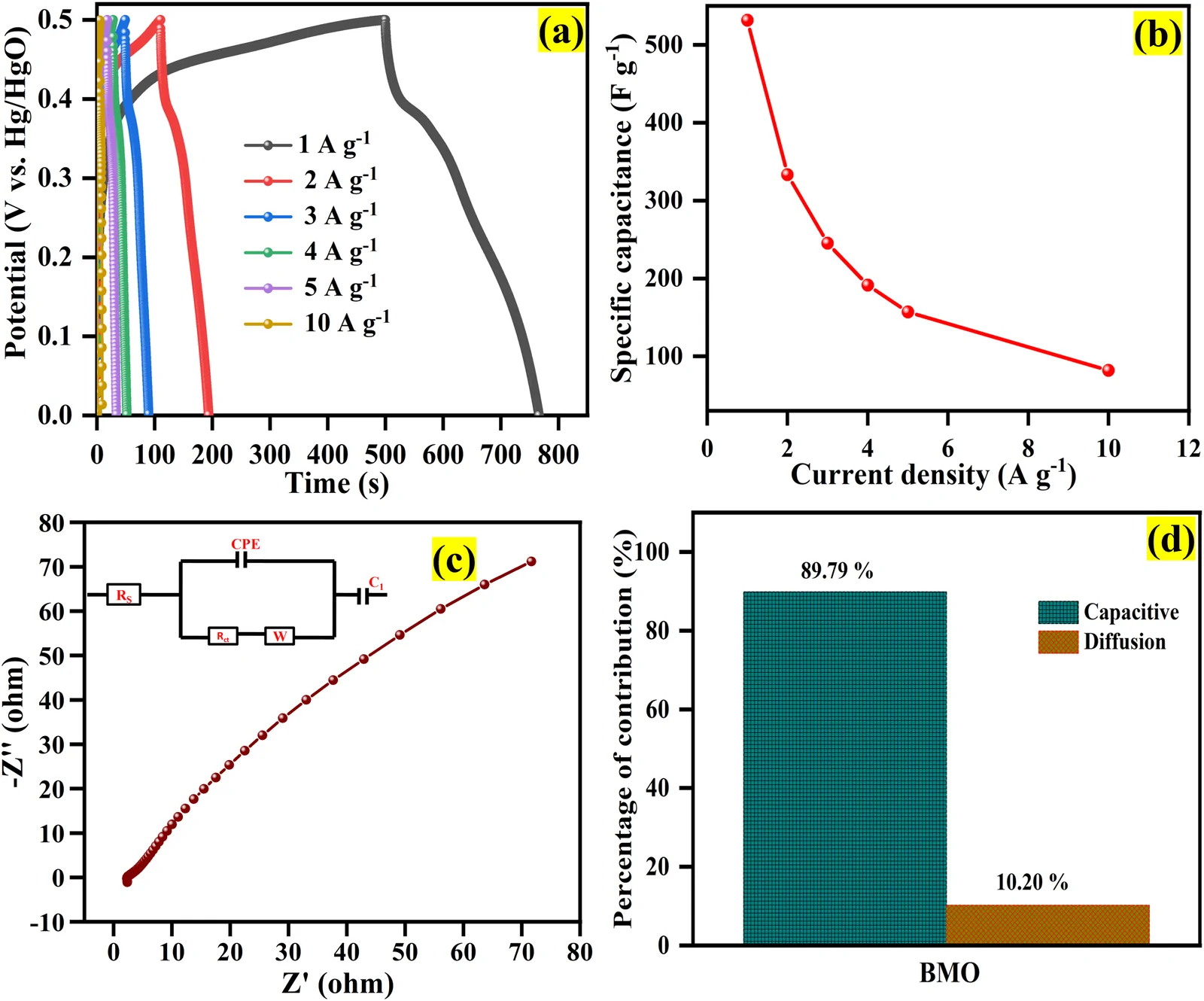
(a) GCD curve, (b) Specific capacitance, (c) EIS, (d) Contribution analysis.

In order to investigate the resistance, ion diffusion and charge-transfer properties of the BMO-15 electrode in the frequency range of 1 Hz to 100 kHz, electrochemical impedance spectroscopy (EIS) was performed as shown in Fig 8(c). The intercept on the Z′ axis in the high frequency region in the Nyquist plot corresponds to the solution resistance (Rs) and the semicircle in the high frequency region corresponds to the charge-transfer resistance (Rct). The values of Rs obtained (0.10 Ω) and Rct (0.50 Ω) indicate low internal resistance and effective electron transfer. The XRD analyses also confirmed the formation of a stable tetragonal scheelite structure with an average crystallite size of 17.05 nm which is consistent with these findings. The nanocrystallinity and uniform lattice structure offer a large number of active sites and provide short ion and electron diffusion pathways, which accelerate the redox reaction and increase the electrochemical performance of the BMO-15 electrode [30,31]. In accordance with the results presented in Fig 8(d), the BMO-15 electrode has 89.79% capacitive controlled charge contribution (q_inner_) and 10.20% diffusion controlled charge contribution (q_outer_). Cycling stability is one of the key requirements for practical supercapacitor applications. The BMO-15 electrode achieved a remarkable electrochemical stability after testing over 10,000 cycles at 6 A g^−1^, and maintained 90% of initial capacitance [32].

### 3.7. Asymmetric BMO-15//AC Device

The activated carbon (AC) was used as the negative electrode and the synthesized BMO-15 as the positive electrode to produce an asymmetric supercapacitor (ASC). The device was electrochemically characterized in 6 M KOH aqueous electrolyte in the potential range of 0–1.4 V. The mass balance between both electrodes was determined by using the following:


$$\begin{array}{c} m_{+}\;/\;m_{-}\;=\;({\Delta V}_{-}\;\times \;C_{-})\;/\;({\Delta V}_{+}\;\times \;C_{+}) \end{array}$$ (15)

In which C₊ and C ₋ are specific capacitance of positive and negative electrodes, respectively, and m₊ and m ₋ are their respective masses, and ΔV₊ and ΔV ₋ are their respective potential windows, respectively. The calculated mass ratio was 1:1, and the total amount of active material was 6 mg. Fig 9(a) presents the CV curves of the ASC at scan rates of 10, 20, 50, 80, and 100 mV s^−1^. The quasi-rectangular profiles with slight redox features further confirm the significant pseudocapacitive contribution of BMO-15 electrode to the charge storage and its excellent performance in the asymmetric configuration. The GCD properties of BMO-15//AC device are shown in Fig 9(b) in the current density range of 1–5 A g^−1^ at 0–1.4 V. The high symmetry charge and discharge curves further validate the excellent electrochemical reversibility of the device. The capacitive charge storage behaviour of BMO-15//AC asymmetric configuration is further verified by the quasi-linear and triangular GCD profiles.The quasi-linear and triangular GCD profiles further confirm the capacitive charge storage behaviour of the BMO-15//AC asymmetric configuration.

**Fig 9:**
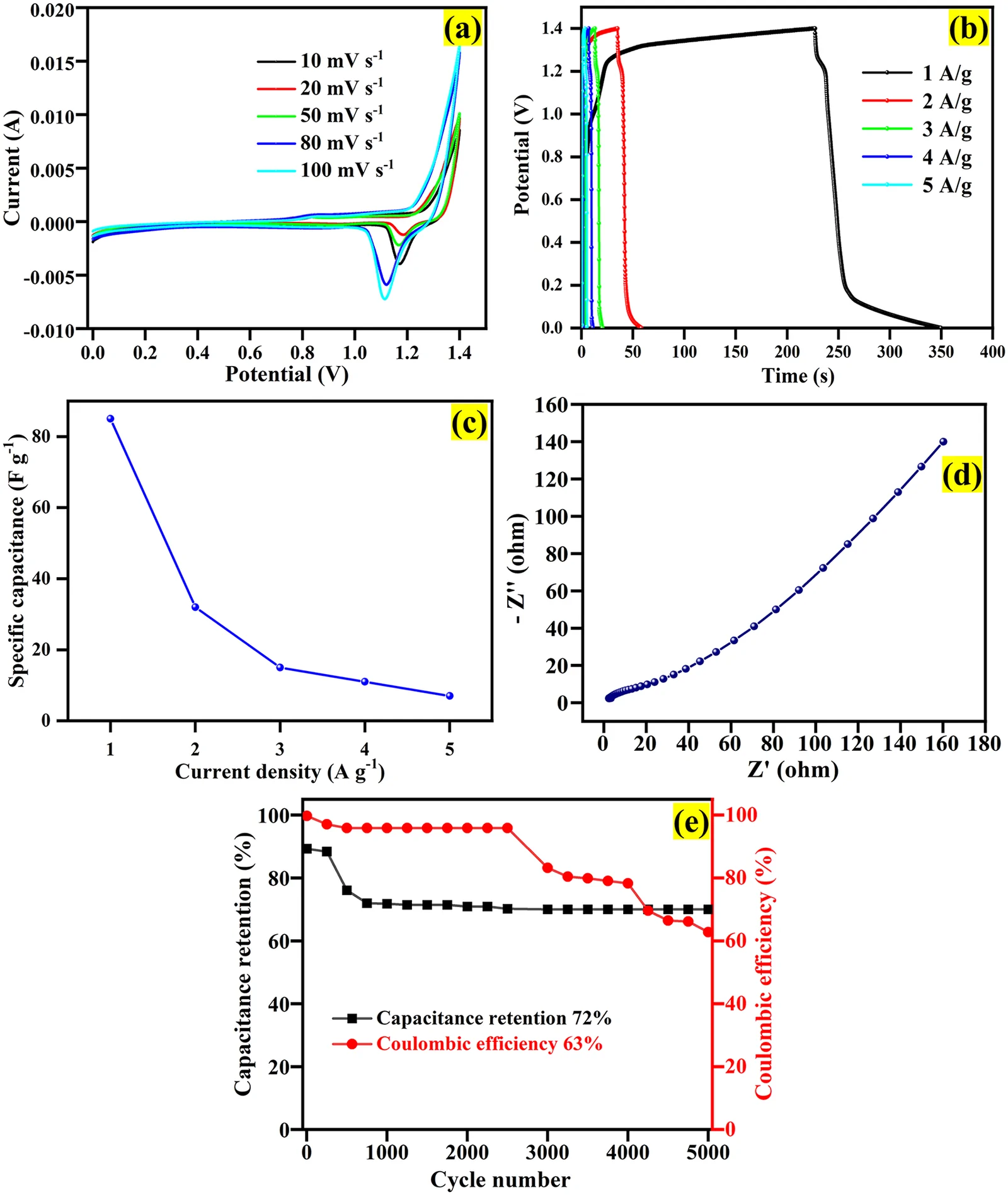
(a) CV graph of the BMO-15//AC device from 10 to 100 mV s^-1^, (b) GCD graph of the BMO-15//AC device from 1 to 5 A g^-1^, (c) Specific capacitance of the BMO-15//AC device at 1–5 A g^-1^, (d) EIS plot of the BMO-15//AC device (e) Coulombic efficiency and capacitance retention graph of the BMO-15//AC device.

Fig 9(c) shows the specific capacitance values of the device, calculated from the discharge curves, of 85, 32, 15, 11, and 7 F g^−1^ at 1, 2, 3, 4, and 5 A g^−1^, respectively. The reduction in specific capacitance with increasing current density is explained by the lack of enough ions available for diffusion at the higher charge/discharge rates, which means that not all of the active sites are being fully utilised.

The Nyquist plot of the BMO-15//AC device is shown in Fig 9(d). The charge transfer resistance (Rct) at the electrode-electrolyte interface and the double-layer capacitance constitute the semicircle in the high-frequency range while the intercept on the real axis corresponds to the solution resistance (Rs). The Rs value for the device is 3.35 Ω and the Rct value is 5.92 Ω, which indicate low internal resistance and efficient charge-transfer kinetics of the device. The Ragone plot (Fig 10) illustrates the relationship between energy density and power density of the BMO-15//AC device. In comparison with other barium nanomaterials, as given in Table 2, the device has a high energy density of 33.05 Wh kg^−1^ and a power density of 1399 W kg^−1^. The long term cycling performance of the device in terms of charge and discharge cycles over 5000 cycles is shown in Fig 9(e). The capacitance of the device remains 72% of the original capacitance, indicating that the structural strength and electrochemical stability of the BMO-15//AC asymmetric configuration is excellent.

**Table 2:** Comparing the power density and energy density for barium materials.

S. No	Compounds	E_d_ (Wh kg^-1^)	P_d_ (W kg^-1^)	Ref.
1	NiMoO_4_	13.2 Wh/kg	1644 W/kg	[33]
2	Sr-doped BaO	32.12 Wh/kg	750 W/kg	[19]
3	Ba-doped SrO	28.68 Wh/kg	250 W/kg	[34]
4	BaMnO_3_	14.85 Wh/kg	225 W/kg	[35]
5	Cu doped Ba(OH)_2_	11.3 Wh/kg	279.7 W/kg	[21]
6	Ba-doped Na_2_ZrO_3_	23.7 Wh/kg	190.8 W/kg	[36]
7	BMO-15	33.05 Wh/kg	1399 W/kg	**This work**

**Fig 10:**
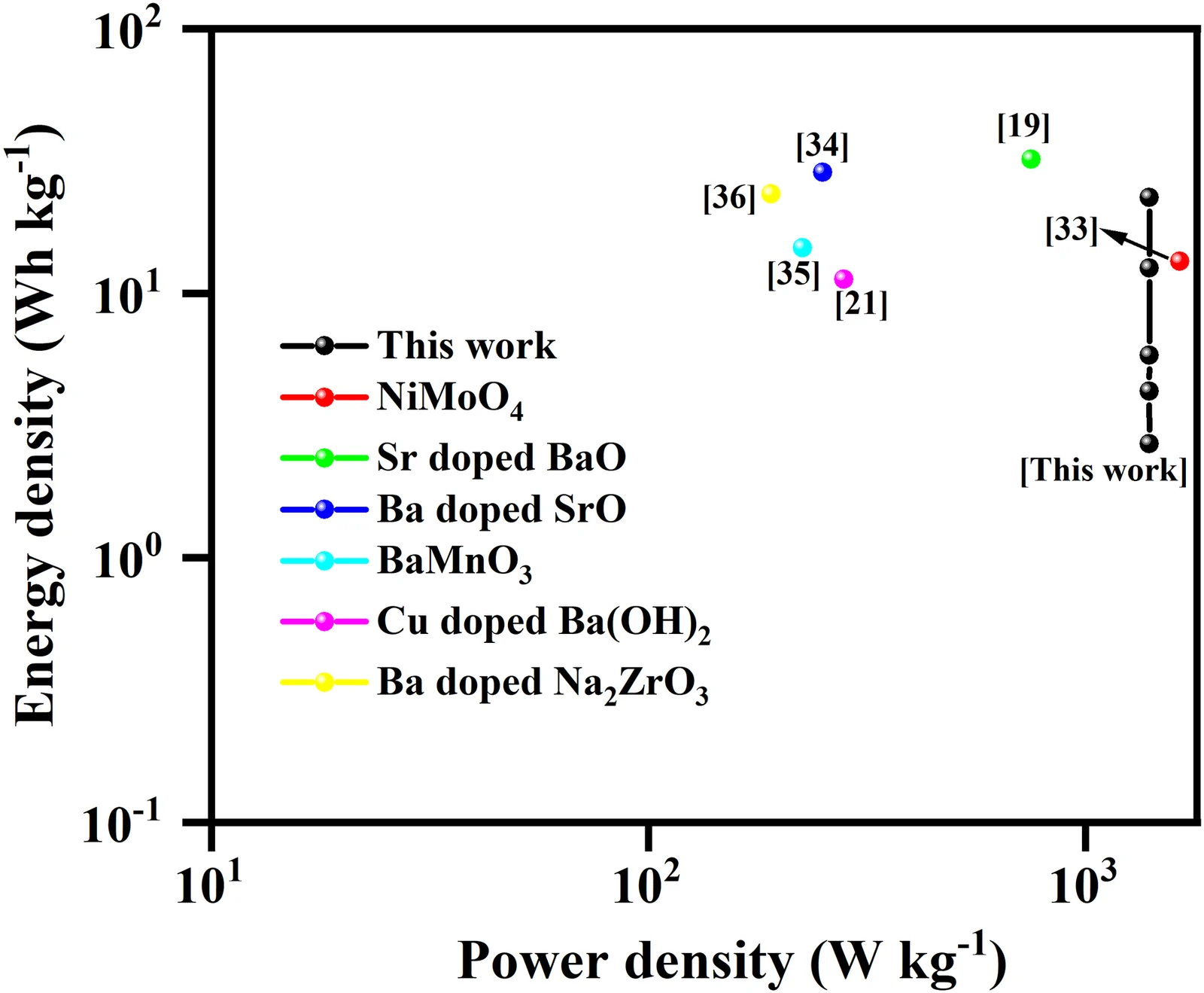
Ragone plot BMO-15//AC device.

The coulombic efficiency of the assembled ASC device was calculated to be approximately 63%. This value may be associated with irreversible faradaic processes, polarisation losses, ion transport limitations within the electrode/electrolyte interface, and partial utilisation of electrochemically active sites during cycling. Furthermore, differences in the charge-storage kinetics of the positive and negative electrodes can contribute to charge imbalance and energy dissipation, resulting in a reduction in the overall coulombic efficiency. Nevertheless, the ASC device exhibits stable electrochemical performance, demonstrating the suitability of BaMoO_4_ nanoparticles for high-performance energy storage applications.

### 3.8. Post-stability

The BMO-15 material was then characterised by XRD and SEM after application in supercapacitors to gain insight into the crystal structure and morphology. The BMO-15 crystal structure had not significantly changed after long-term supercapacitor operation, with very minor changes in the peak intensities as observed in the S8 Fig in S2 File The BMO-15 shape was also well preserved (S9 Fig in S2 File) because of the encapsulating effect of the nanosheets.

## 4. Conclusions

In summary, the BMO have been successfully synthesised by the hydrothermal synthesis times of 5, 10, 15, and 20 h, respectively. The XRD patterns show that a single-phase BMO structure was obtained. The SEM images revealed the nanosheet-like structure of BMO-15. Based on the results of the GCD tests, the specific capacitance of the BMO-15 sample is higher than that of the BMO-5, BMO-10 and BMO-20 samples at a current density of 1 A g^-1^. Additionally, the constructed BMO-15//AC asymmetric supercapacitor achieved a significant energy density of 33.05 Wh kg^-1^ and a power density of 1399 W kg^-1^, demonstrating remarkable long-term stability at 1.4 V, along with impressive cycling stability, maintaining 72% of the original capacitance after 5000 cycles. The capability of the capacitive behaviour could be attributed to the nanosheet structure and BET surface area, while the excellent conductivity of the BMO-15 electrode also benefits the improvement of the capacitive behaviour. The results indicate the BMO-15 hybrid as a promising electrode material for advanced energy storage systems.

## Supporting information

SI File Experimental Results* Original Datas. (ZIP)

S2 File S1 Fig. (a, b) FESEM images of BMO-5, (c) EDS spectrum of BMO-5, (d-g) Element mapping images of Ba, Mo, O.* Suppl Fig only. S2 Fig. (a, b) FESEM images of BMO-10, (c) EDS spectrum of BMO-10, (d-g) Element mapping images of Ba, Mo, O. * Suppl Fig only. S3 Fig. (a, b) FESEM images of BMO-20, (c) EDS spectrum of BMO-20, (d-g) Element mapping images of Ba, Mo, O. * Suppl Fig only. S4 Fig. BMO-5 graphs of (a) CV, (b) GCD, (c) specific capacity at different current rates, (d) Nyquist plots. * Suppl Fig only. S5 Fig. BMO-10 graphs of (a) CV, (b) GCD, (c) specific capacity at different current rates, (d) Nyquist plots. * Suppl Fig only. S6 Fig. BMO-20 graphs of (a) CV, (b) GCD, (c) specific capacity at different current rates, (d) Nyquist plots. * Suppl Fig only. S7 Fig. CV plots at 100 mV s^-1^ in different voltages. * Suppl Fig only. S8 Fig. XRD of BMO-15 before and after cycling. * Suppl Fig only. S9 Fig. (a, b) SEM pictures of BMO-15 after cycling. * Suppl Fig only.
(ZIP)
