# Supplementary material for: Hydrothermal reaction time-dependent morphology and electrochemical performance of BaMoO4 for asymmetric supercapacitors
Source: PLoS One. 2026 Sep 29;21(9):e0354958. doi: 10.1371/journal.pone.0354958 (PMC13623147; doi:10.1371/journal.pone.0354958)
Supplement: Supplementary file 1 [file pone.0354958.s001.zip › Original datas/SEM/Befor and After SEM - 15 h/pow dex Project 1_Site 1_2026-05-22_14-00-40.docx]

Click here to enter text.

| Element | Line Type | Apparent Concentration | k Ratio | Wt% | Wt% Sigma | Atomic % | Standard Label |
| --- | --- | --- | --- | --- | --- | --- | --- |
| O | K series | 2.85 | 0.00961 | 16.23 | 0.77 | 59.07 | SiO2 |
| Mo | L series | 3.85 | 0.03852 | 29.53 | 1.12 | 17.93 | Mo |
| Ba | L series | 7.76 | 0.07266 | 54.24 | 1.15 | 23.00 | BaF2 |
| Total: |  |  |  | 100.00 |  | 100.00 |  |
