# Supplementary material for: Hydrothermal reaction time-dependent morphology and electrochemical performance of BaMoO4 for asymmetric supercapacitors
Source: PLoS One. 2026 Sep 29;21(9):e0354958. doi: 10.1371/journal.pone.0354958 (PMC13623147; doi:10.1371/journal.pone.0354958)
Supplement: Supplementary file 1 [file pone.0354958.s001.zip › Original datas/SEM/Befor and After SEM - 15 h/pow map Project 1_Site 1_2026-05-22_14-04-43.docx]

Click here to enter text.
